# Eugenol alleviated breast precancerous lesions through HER2/PI3K-AKT pathway-induced cell apoptosis and S-phase arrest

**DOI:** 10.18632/oncotarget.17626

**Published:** 2017-05-05

**Authors:** Min Ma, Yi Ma, Gui-Juan Zhang, Rui Liao, Xue-Feng Jiang, Xian-Xin Yan, Feng-Jie Bie, Xiao-Bo Li, Yan-Hong Lv

**Affiliations:** ^1^ College of Traditional Chinese Medicine, Institute of Integrated Traditional Chinese and Western Medicine, Jinan University, Guangzhou 510632, Guangdong Province, China; ^2^ Institute of Biomedicine, Department of Cellular Biology, Key Laboratory of Bioengineering Medicine of Guangdong Province, Jinan University, Guangzhou 510632, Guangdong Province, China; ^3^ The First Affiliated Hospital of Jinan University, Guangzhou 510632, Guangdong Province, China

**Keywords:** eugenol, breast precancerous lesion, HER2/PI3K-AKT, external use, cell apoptosis

## Abstract

Eugenol can be separated from the oil extract of clove bud, and has many pharmacological functions such as anticancer and transdermal absorption. HER2/PI3K-AKT is a key signaling pathway in the development of breast cancer. In this study, 80 μM eugenol could significantly inhibit the proliferation of HER-2 positive MCF-10AT cells and the inhibition rate was up to 32.8%, but had no obvious inhibitory effect on MCF-7 and MCF-10A cells with HER2 weak expression. Eugenol also significantly induced human breast precancerous lesion MCF-10AT cell apoptosis and cell cycle S-phase arrest, but the biological effects nearly disappeared after HER2 over-expression through transfecting pcDNA3.1-HER2. In MCF-10AT cells treated by 180 μM eugenol, the protein expressions of HER2, AKT, PDK1, p85, Bcl2, NF-κB, Bad and Cyclin D1 were decreased and the decreased rates were respectively 63.0%, 60.0%, 52.9%, 62.9%, 37.1%, 47.2%, 61.7%, 59.1%, while the p21, p27 and Bax expression were increased by 4.48-, 4.76- and 2.57-fold respectively. In the rat models of breast precancerous lesion, 1 mg eugenol for external use significantly inhibited the progress of breast precancerous lesion and the occurrence rate of breast precancerous lesions and invasive carcinomas was decreased by about 30.5%. Furthermore eugenol for external (1 mg) markedly decreased the protein expressions of HER2 (62.9%), AKT (58.6%), PDK1 (56.4%), p85 (54.3%), Bcl2 (59.3%), NF-κB (65.7%), Bad (64.0%), Cyclin D1 (43.0%), while p21, p27 and Bax protein expressions were respectively increased 1.83-, 2.52- and 2.51-fold. The results showed eugenol could significantly inhibit the development of breast precancerous lesions by blocking HER2/PI3K-AKT signaling network. So eugenol may be a promising external drug for breast precancerous lesions.

## INTRODUCTION

Breast cancer accounts for about 29% of female malignancies, and breast cancer mortality rates rank the first in female cancer patients. The development of breast cancer goes through the continuous process containing normal, hyperplasia, atypical hyperplasia and carcinoma. Precancerous lesion, the essential stage of breast cancer progression, can be blocked or reversed via specific drug intervention. Thereby, the incidence of breast cancer can be effectively decreased due to the block for tumor development process [1].

Probability of malignant tumors resulted from precancerous lesions was more than 20%, notably, breast precancerous lesions mainly including atypical hyperplasia (atypical ductal or lobular hyperplasia), ductal carcinoma *in situ*, ductal carcinoma, papillary tumor, resulted in approximately 50% probability of breast cancer occurring. Hoogerbruggeet et al found that the incidence rates of precancerous lesion, atypical lobular hyperplasia, atypical ductal hyperplasia, small leaf *in situ*, and ductal carcinoma *in situ* were respectively 57%, 37%, 39%, 25% and 15% in the susceptible population of breast cancer. Moreover, the incidence with high risk of breast cancer was significantly increased after 40 years of age [2].

Currently, the relationship between precancerous lesion and breast cancer, and the regular pattern of continuously developmental process in breast cancer are being studied by more and more researchers. It was found that breast hyperplasia, precancerous lesion and invasive cancer could appear simultaneously in the histopathological sections of breast cancer, and the relationship between precancerous lesion and breast cancer was closer than breast hyperplasia, which also revealed that precancerous lesion was an important early-stage in the development of breast cancer. On the contrary, if breast precancerous lesions can be kept from the pathogenic factors or intervened effectively by therapeutics, it may stay in a stable state for a long time and even get good reverse recovery. HER2/PI3K-AKT signal transduction pathway plays an important role in breast cancer occurrence and the signal transduction network formed by HER2/PI3K-AKT signaling pathway is closely related to the occurrence, development and treatment of breast cancer [3, 4]. However, the relationship between breast precancerous lesions and HER2/PI3K-AKT signal transduction pathway is not clear so far.

Studies have shown that tamoxifen (Tam) can be used for the treatment of breast precancerous lesions and early prevention of breast cancer. There is no significant difference between the clinical curative effect of Tam external and oral use. Moreover, Tam external use has a function of sustained release properties compared with oral use. In addition, some studies also showed that Tam external use can obviously inhibit breast precancerous lesions, reduce the blood flow rate of local microcirculation, adjust the levels of pituitary and sex hormone, decrease oxygen free radicals and accelerate metabolism so as to further block the development of breast cancer through acting as estrogen receptor antagonist [5, 6]. However, Tam is prone to cause endocrine disorders, liver and kidney damage [7, 8].

Eugenol (Eug) [4-allyl (-2-mthoxyphenol), molecular formula: C_10_H_12_O_2_], a phenolic natural compound available in honey and in the essential oils of different spices such as clove, bay leaves and cinnamon leaf, has been exploited for various medicinal applications. Eug has a variety of pharmacological activities such as analgesic, antiseptic, antioxidant, antibacterial, anticancer, anti-inflammatory and so on [9]. Al-Sharifet et al proved that Eug could trigger apoptosis in breast cancer cells through E2F1/survivin down-regulation and also inhibit the growth of breast cancer tissues *in vitro* [10]. Kumar et al indicated clove had significant growth inhibition effect on MCF-7 cell line of breast cancer by brine shrimp lethality test (BSLT) and *3-*(*4,5*)-*dimethylthiahiazo*(-*z-y1*)-*3,5-di-phenytetrazoliumromide* (MTT) determination [11]. Hussain et al showed that there was a synergistic effect between Eug and gemcitabine (an anticancer drug), which could improve the therapeutic index of cancer, and Eug could significantly reduced the expression of Bcl2, COX-2 and IL-1β [12]. These results indicate that Eug can resist cancer by inducing apoptosis and its anti-inflammatory properties.

In this study, BT-474, MCF-7, MCF-10A, MCF-10AT cells and model rats of breast precancerous lesions were treated with different concentrations of Eug to study the biological effects of Eug on anti-breast precancerous lesions and its mechanism of inducing apoptosis and cell cycle arrest by affecting HER2/PI3K-AKT signaling pathway.

## RESULTS

### Eug might significantly inhibit cells proliferation through blocking the target HER2

**Figure 1:**
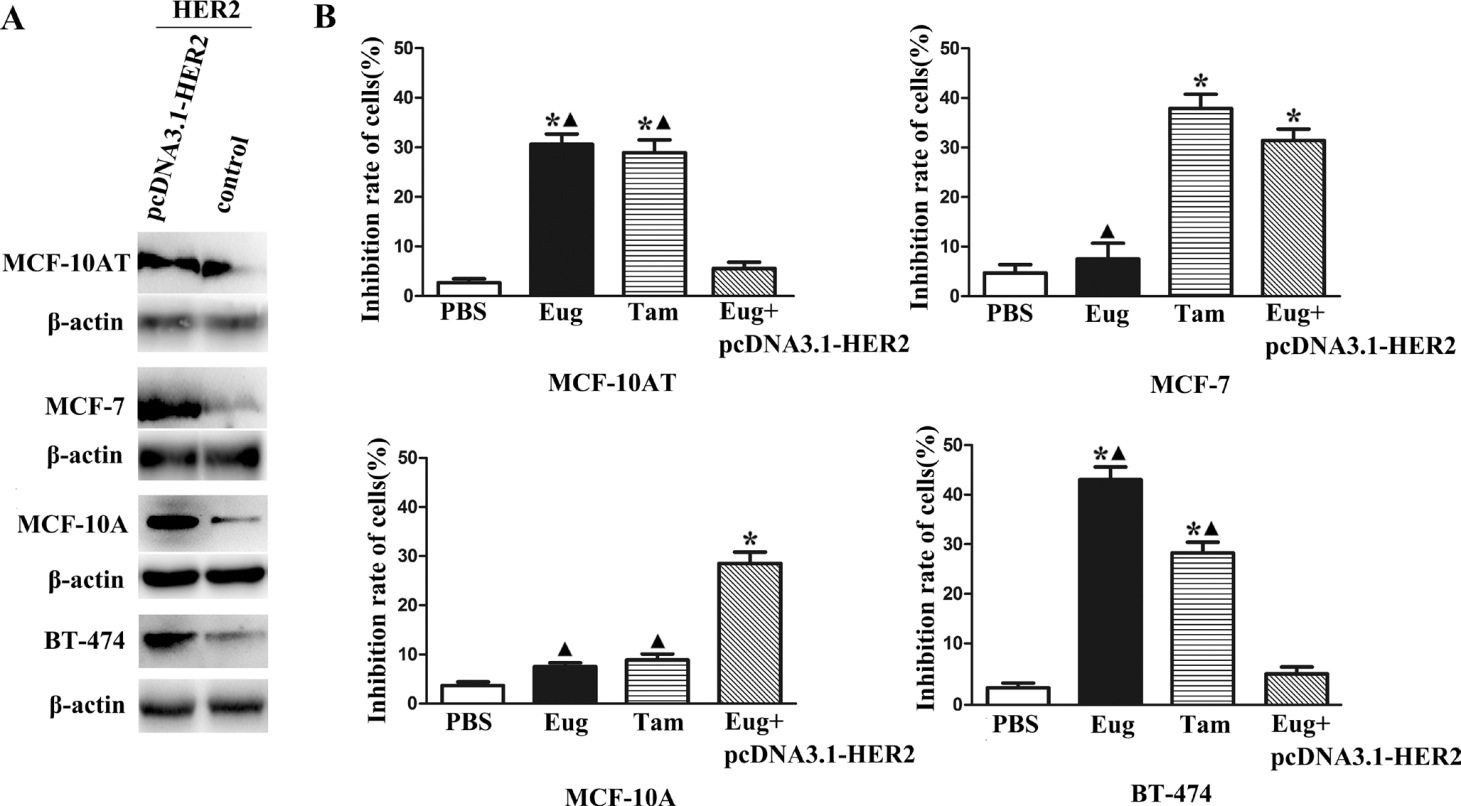
Overexpression of HER2 in BT-474, MCF-10AT, MCF-7 and MCF-10A cells (**A**) and inhibitory effects on proliferation of eugenol (Eug), tamoxifen (Tam) on HER2 non-overexpressing and overexpressing BT-474, MCF-10AT, MCF-7 and MCF-10A cells (Eug+pcDNA3.1-HER2) (**B**). *P < 0.05, Eug, Tam or (Eug+pcDNA3.1-HER2) versus PBS; ▲*P* < 0.05, Eug, Tam or PBS versus (Eug+pcDNA3.1-HER2) (Scheff´e test, *n* = 3).

The BT-474, MCF-10AT, MCF-7 and MCF-10A cells in logarithmic growth phase were treated by different concentrations of Eug (40, 80, 120, 160, 200 and 240 μM) for 24 h, and the cells growth inhibition rates were detected. After the cells were treated by different concentrations of Eug for 24 h, MCF-10AT cells growth could be significantly inhibited. The 50% inhibiting concentration (IC50) of Eug on HER-2 positive MCF-10AT cells was 160.9 μM. In BT-474, MCF-10AT, MCF-7 and MCF-10A cells with HER2 over-expression through transfecting plasmid pcDNA3.1-HER2, the protein expression levels of HER2 were respectively 3.14-, 2.37-, 4.32- and 3.53-fold of the corresponding controls (Figure 1A). As shown in Figure 1B, the inhibition rates of 80 μM Eug and Tam on MCF-10AT were 32.8% and 28.9% respectively, which were significantly higher than PBS-treated group (blank control). The inhibition rate of Eug (80 μM) in MCF-10AT cells with HER2 over-expression was only 6.7% and showed no significant difference compared with PBS-treated group (Figure 1B). Eug (80 μM) had no obvious inhibitory effect on MCF-7 and MCF-10A cells with HER2 weak expression, but the inhibition rates of Eug significantly increased in MCF-7 and MCF-10A cells with HER2 over-expression (Figure 1B). The inhibition rate of 80 μM Eug for BT-474 cells with HER2 high expression was up to 43.3%, but the inhibition effect basically disappeared in BT-474 cells with HER2 over-expression (Figure 1B). These results showed that Eug might significantly inhibit MCF-10AT and BT-474 cells proliferation through blocking the target HER2.

### Eug could significantly promote MCF-10AT cells apoptosis

**Figure 2:**
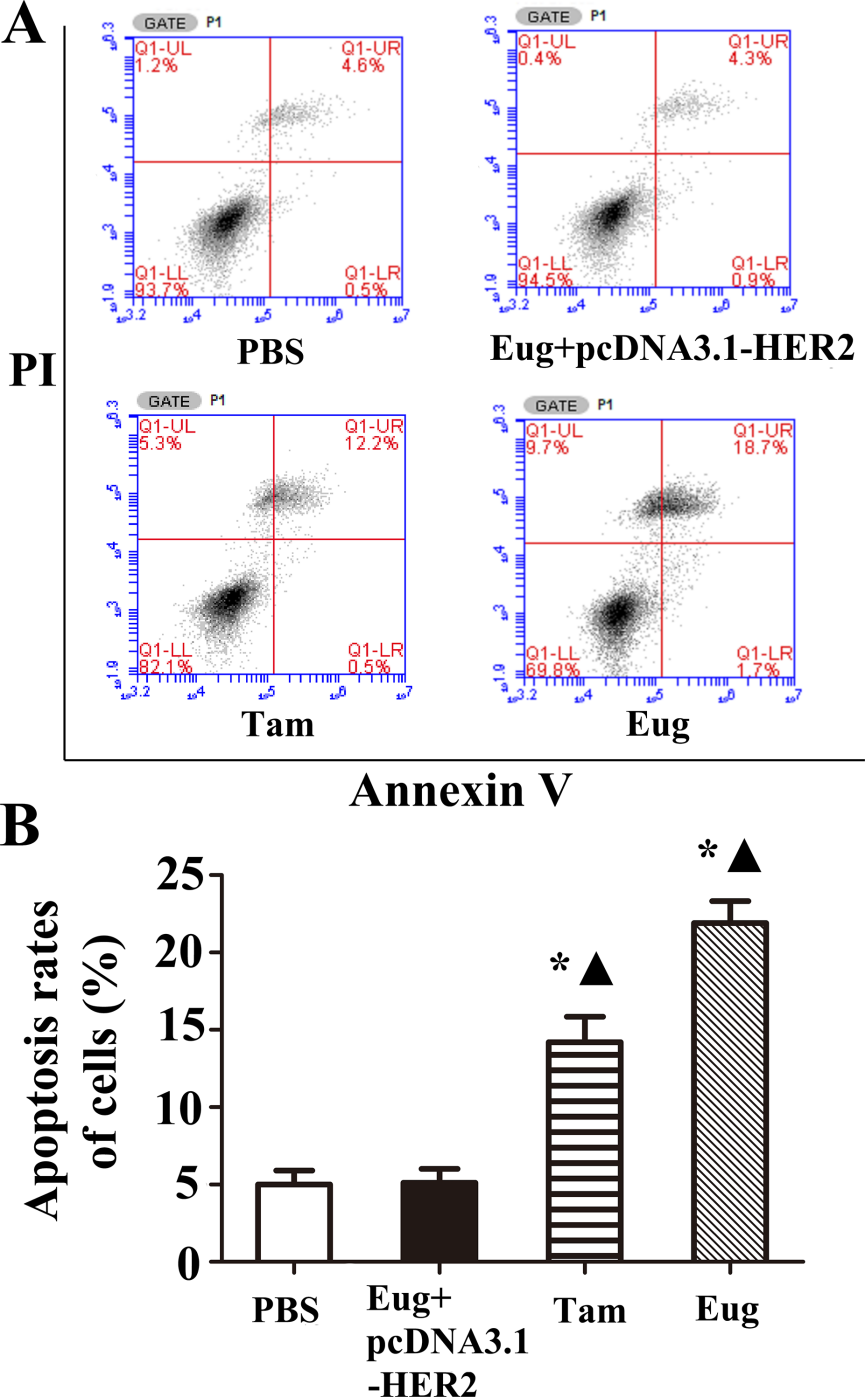
Eugenol (Eug) increased apoptosis for human breast precancerous lesion MCF-10AT cells (**A**) Representing assays of tamoxifen (Tam) and Eug inducing apoptosis for HER2 non-overexpressing and overexpressing (Eug+pcDNA3.1-HER2) MCF-10AT cells by Annexin V-FITC/PI double staining-flow cytometry. (**B**) Statistics and data analysis of apoptosis in MCF-10AT cells in (A). PBS was taken as the control group (Vehicle). *P < 0.05, Eug, Tam or (Eug+pcDNA3.1-HER2) versus PBS; ▲*P* < 0.05, Eug, Tam or PBS versus (Eug+pcDNA3.1-HER2) (Scheff´e test, *n* = 3).

MCF-10AT cells and MCF-10AT cells with HER2 over-expression in logarithmic phase inoculated in 6-well plate were treated with PBS, Tam or Eug (180 μM) for 24 h. Then the effects of Eug on the apoptosis of MCF-10AT cells were determined by AnnexinV-FITC/PI double staining-flow cytometry. The experimental results showed that MCF-10AT cell apoptosis rates were respectively 5.00%, 20.73% and 11.50% in PBS-, Eug-, and Tam-treated groups (Figure 2A and 2B). The apoptosis rate of PBS-treated group showed no significant difference compared with Eug-treated MCF-10A cells with HER2 over-expression group. MCF-10AT cells apoptosis rates of Eug- and Tam-treated groups were obviously higher than that of PBS-treated group, suggesting that Eug and Tam could significantly promote MCF-10AT cells apoptosis. The apoptosis-promoting effect of Eug on MCF-10AT cells disappeared after overexpressing HER2 (Figure 2A and 2B).

### Eug could significantly induce S-phase cell cycle arrest in breast precancerous lesion MCF-10AT cells

**Figure 3:**
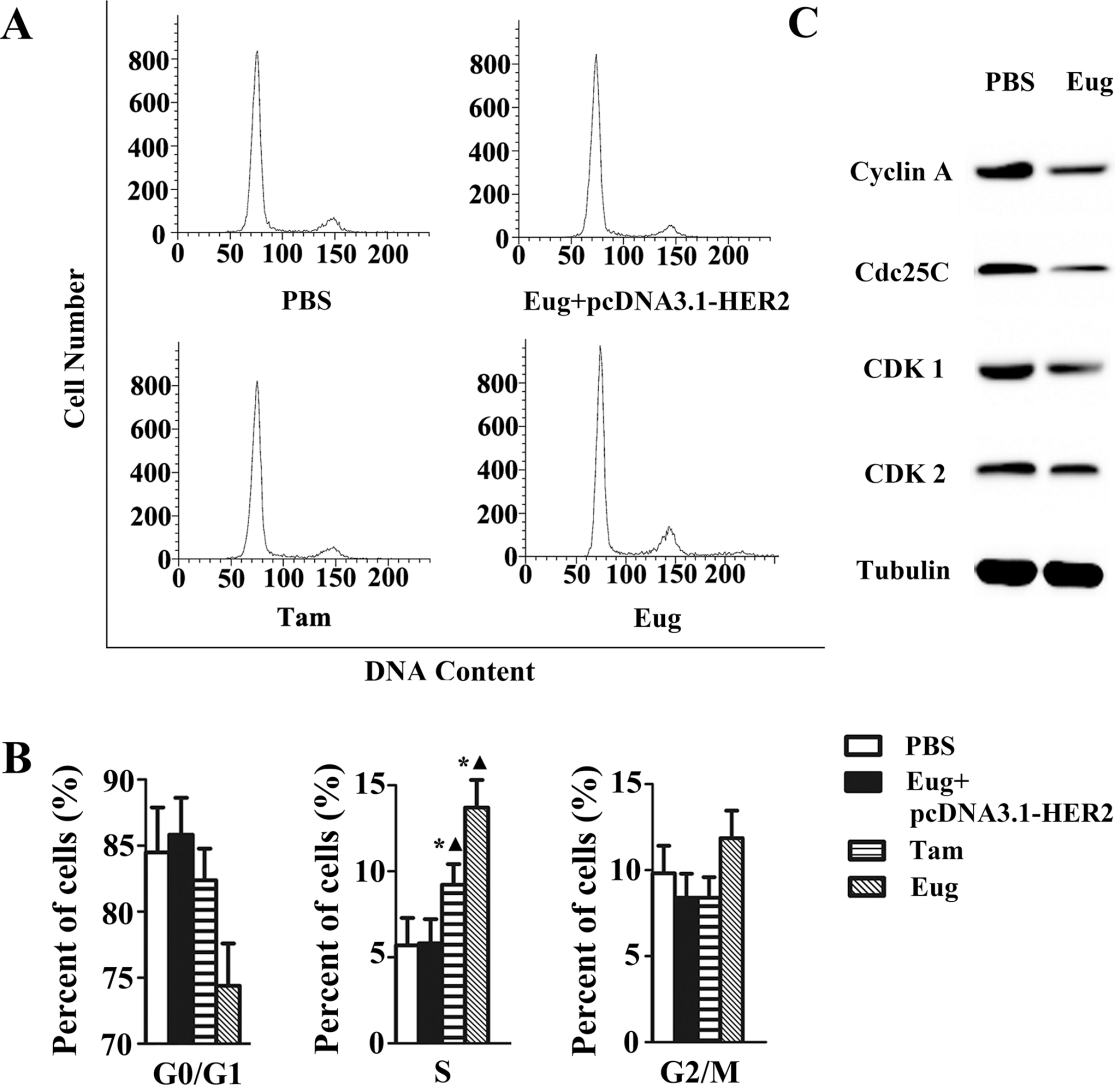
Eugenol (Eug) induced S-phase cell cycle arrest in HER2 non-overexpressing MCF-10AT cells (**A**) Flow cytometry assays of the cells cycle for PBS-, Eug- or tamoxifen (Tam)-treated MCF-10AT cells and Eug-treated HER2 overexpressing MCF-10AT cells (Eug+pcDNA3.1-HER2). (**B**) Statistics and data analysis of MCF-10AT cells cycle distribution in (A). (**C**) Western-blot assays of CDK1, CDK2, Cdc25C and Cyclin A which are mainly involved in driving the transition from S to G2/M phase. *P < 0.05, Eug, Tam or (Eug+pcDNA3.1-HER2) versus PBS; ▲*P* < 0.05, Eug, Tam or PBS versus (Eug+pcDNA3.1-HER2) (Scheff´*e* test, *n* = 3).

MCF-10AT cells and MCF-10AT cells with HER2 over-expression in logarithmic growth phase inoculated in 6-well plate with 1 × 10^5^ cells/well were treated with PBS, Tam or Eug (180 μM) for 24 h. The PI single staining-flow cytometry was applied to determine the effects of Eug on cell cycle of MCF-10AT cells. As shown in Figure 3A and 3B, the results showed that the cell percentages of G0/G1, S and G2/M phases were 74.29%, 13.91% and 11.80% respectively in Eug-treated group, whereas the cell percentages of G0/G1, S and G2/M phases were 84.49%, 5.69%, 9.84% in PBS-treated group. The analysis showed that MCF-10AT cells remaining in S phase were significantly increased by 8.22% after the treatment of Eug for 24 h, correspondingly, the cells remaining in G0/G1 phase and G2/M phase were reduced by 8.24%, furthermore the decrease of the cells remaining in G0/G1 phase was main and the number of G2/M phase cells has no significant changes (Figure 3A and 3B). However, the biological effect of Eug *inducing S-phase* cell cycle *arrest basically disappeared in* MCF-10AT cells with HER2 over-expression. This indicated that Eug could block the transformation of cells from S to G2/M phase, thereby inhibiting cell proliferation, compared with Eug, Tam showed a weaker ability in reducing S-phase arrest (3.53%). Furthermore, as shown in Figure 3C, the protein expression of CDK1, CDK2, Cdc25C and Cyclin A which are mainly involved in driving the transition from S to G2/M phase were markedly reduced in Eug-treated HER2 non-overexpressing MCF-10AT cells. The results showed that Eug could significantly induce S-phase cell cycle arrest in MCF-10AT cells, and the effect was obviously stronger than Tam.

### Effects of Eug on the key protein molecules expressions of HER2/PI3K-AKT signaling pathway in MCF-10AT cells

**Figure 4:**
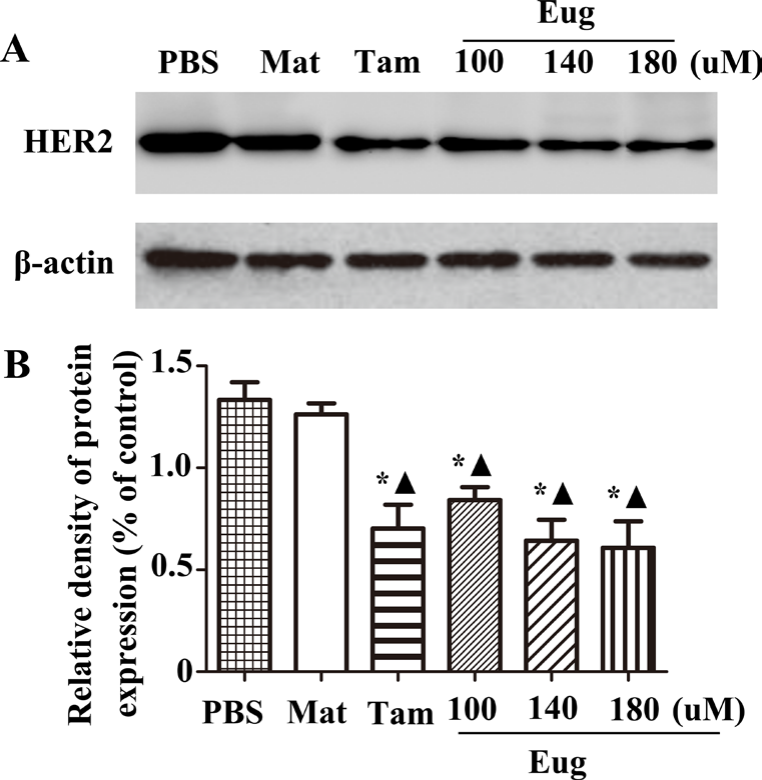
Eugenol (Eug) significantly inhibited the protein expression of HER2 (**A**) Western-blot assays of HER2 expression at 24 h after PBS, blank matrix (Mat), Eug or tamoxifen (Tam) treatment in MCF-10AT cells. (**B**) Relative expression assays for HER2 in (A). PBS and Mat were taken as the blank control groups. *P < 0.05, Eug (100, 140 or 180 μM), Tam or Mat versus PBS; ▲*P* < 0.05, Eug (100, 140 or 180 μM), Tam or PBS versus Mat (Scheff´e test, *n* = 3).

MCF-10AT cells in logarithmic growth phase inoculated in a 96-well plate were treated with Eug (100, 140 or 180 μM), Tam (180 μM), PBS and the blank matrix (Mat, 40 g stearic acid was heated in a water bath, melted and cooled down to 80°C.100 g KOH water solution and glycerinum were added and stirred constantly, then the appropriate amount of distilled water was added and the volume was metered to 1000 ml, so as to obtain the blank matrix, the different doses of Eug were added into Mat, thoroughly mixed and cooled down to room temperature, so as to obtain the Eug cream) for 24 h. The experimental results showed that HER2 protein expression almost showed no difference between the PBS-treated group and Mat-treated group. Compared with PBS-treated group or Mat-treated group, HER2 protein expression was significantly decreased in Tam-treated group and different concentrations of Eug-treated groups, and Eug had a dose-dependent effect. HER2 protein expression was lowest in 180 μM Eug-treated group, and HER2 protein expression was decreased by about 51.1% (Figure 4A and 4B).

**Figure 5:**
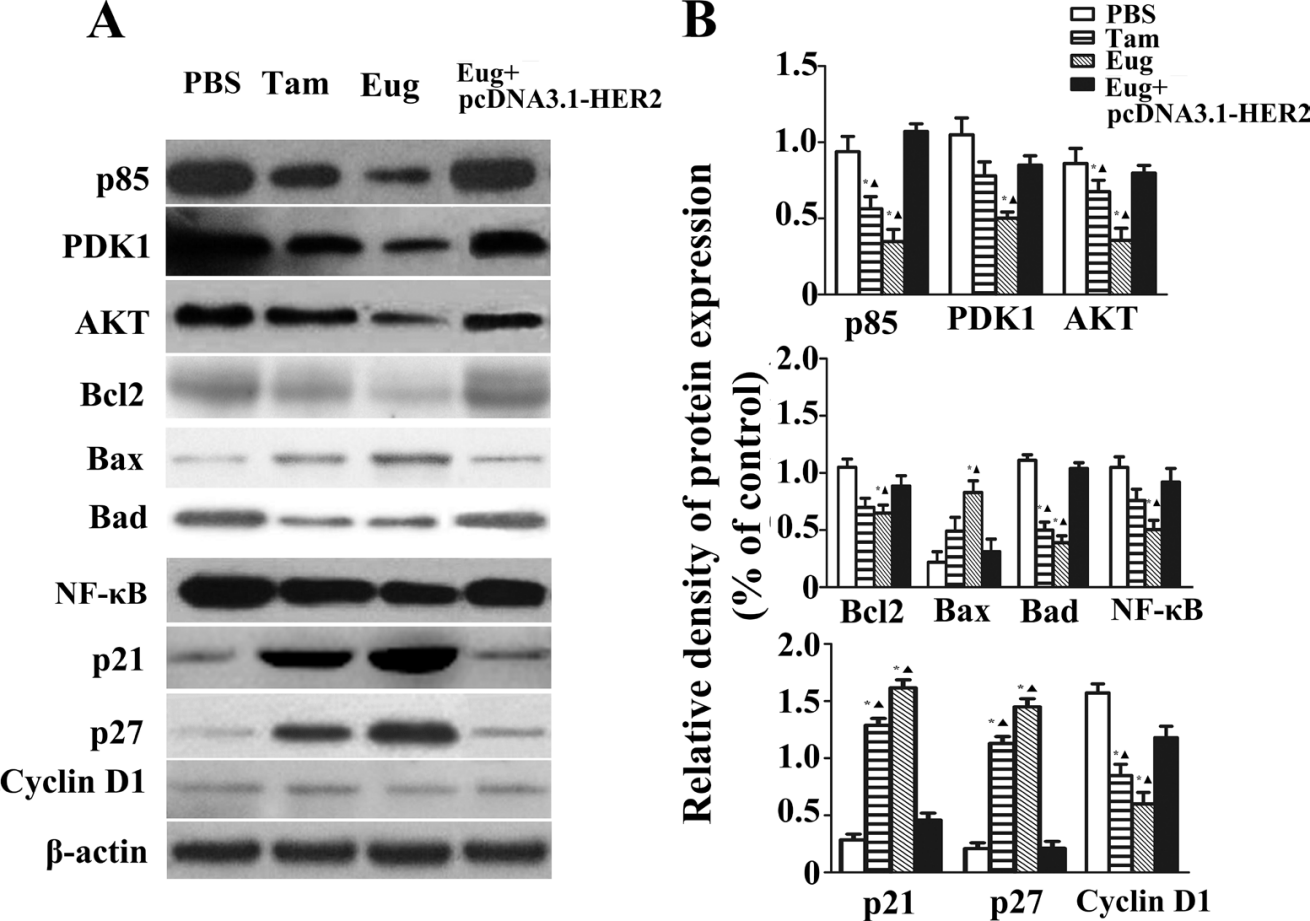
Effect of eugenol (Eug) on the expressions of key proteins P85, PDK1, AKT, Bcl2, Bax, Bad, NF-κB, p21, p27 and Cyclin D1 in HER2/PI3K-AKT signaling pathways in MCF-10AT cells (**A**) Western-blot assays of key protein expressions at 24 h in PBS-, Eug- or tamoxifen (Tam)- treated MCF-10AT cells and Eug-treated HER2 overexpressing MCF-10AT cells (Eug+pcDNA3.1-HER2). (**B**) Relative expression assays for P85, PDK1, AKT, Bcl2, Bax, Bad, NF-κB, p21, p27 and Cyclin D1 in (A). *P < 0.05, Eug, Tam or (Eug+pcDNA3.1-HER2) versus PBS; ▲*P* < 0.05, Eug, Tam or PBS versus (Eug+pcDNA3.1-HER2) (Scheff´e test, *n* = 3).

As shown in Figure 5, compared with the PBS-treated group, the expressions of PDK1, p85 and AKT proteins in MCF-10AT cells were also respectively decreased by 52.9%, 62.9% and 60% in 180 μM Eug-treated group, and the effects were stronger than Tam-treated group. In Eug-treated group the expressions of apoptosis-related factors NF-κB, Bad and Bcl-2 were also respectively decreased by 47.2%, 61.7% and 37.1%, while the Bax protein expression was increased by 2.57-fold (Figure 5A and 5B).

Compared with PBS-treated group, the expressions of cell cycle-associated protein cyclin D1, P21 and p27 were significantly changed in 180 μM Tam- and Eug-treated groups. In 180 μM Eug-treated group the expression of cyclin D1 was decreased by 59.1%, while P21 and p27 protein expression was significantly increased by 4.48-, 4.76-fold, respectively (Figure 5A and 5B).

Eug might induce S-phase arrest and promote MCF-10AT apoptosis through blocking HER2/PI3K-AKT signaling transduction by firstly decreasing HER2 protein expression and further regulating its downstream key signaling molecules expressions in HER2/PI3K-AKT signaling pathway.

### Eug cream had effective therapeutic intervention effects on breast precancerous lesion model rats

#### Breast tissue morphological observation

**Figure 6:**
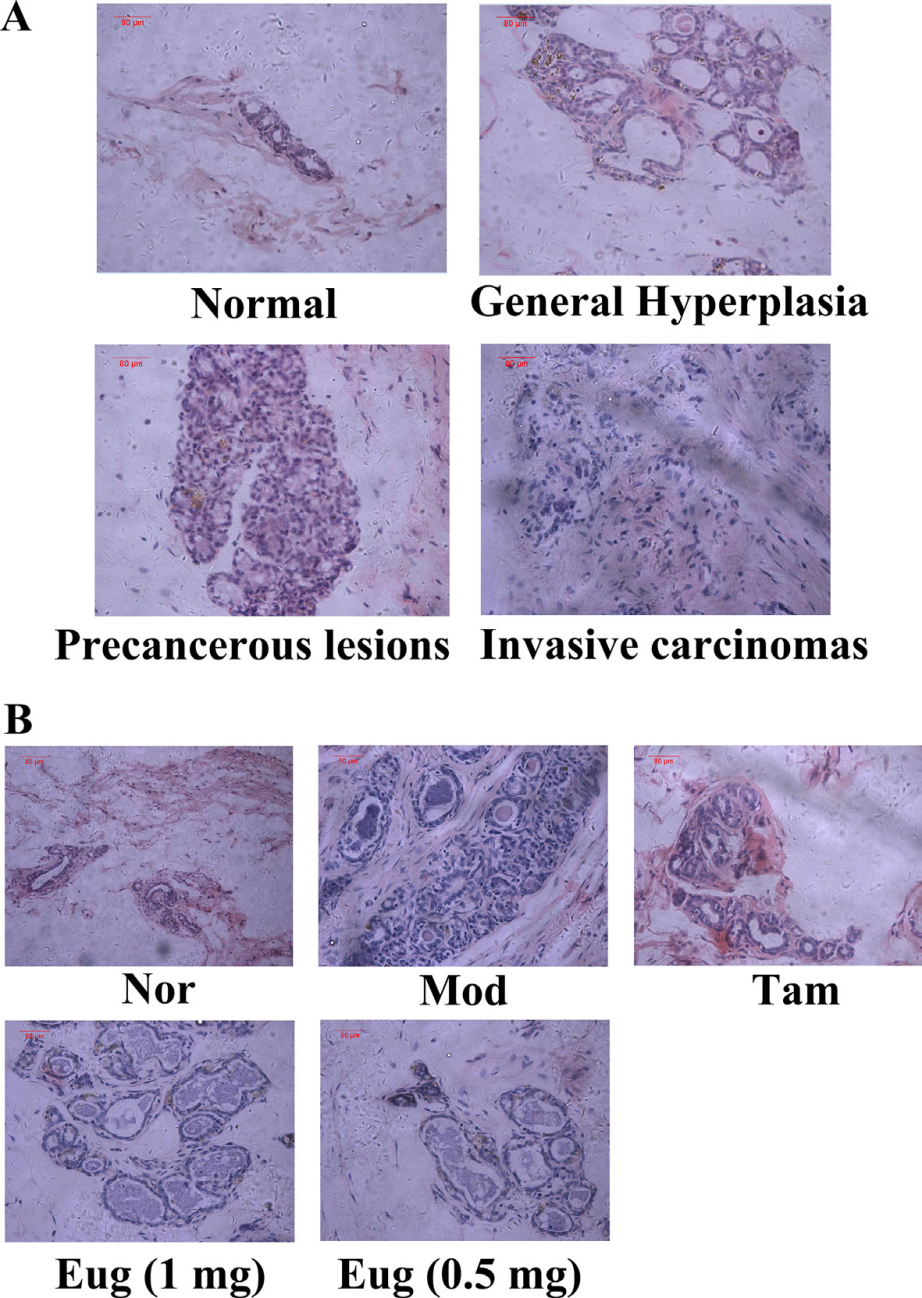
Breast tissue morphological assay for rats and eugenol (Eug) decreased the occurrence of breast precancerous lesions and invasive carcinomas in breast precancerous lesion model rats (**A**) Rats breast tissue morphological detection in the process from normal (Nor), general hyperplasia, precancerous lesions to invasive carcinomas. (**B**) Breast tissue morphological assay for normal (Nor), tamoxifen (Tam, 1 mg)-, low (0.5 mg) or high (1 mg) dose Eug-treated breast precancerous lesion model rats after treatment for 14 weeks.

In light of the pathomorphological characteristics, the precancerous lesion and invasive carcinoma did not occur in the normal blank control group. But varying degrees of precancerous lesions and invasive carcinomas occurred in the disease model group, Tam-, high dose Eug cream- and low dose Eug-treated model rats groups (Figure 6A and 6B). The pathomorphology of the disease model group showed typical precancerous lesion characteristics, which were significantly different from the normal blank control group, suggesting that DMBA in combination with estrogen and progesterone could successfully replicate breast precancerous lesions rat model (Figure 6A and 6B). After being treated by Tam and Eug creams for 14 weeks, compared with the disease model group, the occurrence and development of precancerous lesions and invasive carcinomas were decreased, and the occurrence rates of breast precancerous lesions and invasive carcinomas were respectively decreased by about 40.9%–30.5% and 27.7% in Tam-, high dose Eug- and low dose Eug-treated groups (Table 1, Figure 6B).

**Table 1:** Statistical analysis of the pathological changes of the breast tissues in normal or breast precancerous lesion model rats without treatment or continuously treated with tamoxifen (Tam, 1 mg), low (0.5 mg) or high (1 mg) dose eugenol (Eug) for 14 weeks

Groups	Breast number	No hyperplasia	General hyperplasia	Precancerous lesions	Invasive carcinomas
Normal	144	126▲	18▲	0▲	0▲
Model	144	0*	6*	132*	6*
Tam	144	18*▲	47*▲	78*▲	1*▲
Eug (1 mg)	144	11*▲	39*▲	92*▲	2*▲
Eug (0.5 mg)	144	9*▲	37*▲	94*▲	4*▲

There are 12 rats in every group and every rat has six pairs of nipples. *P < 0.05, Model, Eug (0.5 or 1 mg)- or Tam-treated rats versus normal control rats; ▲*P* < 0.05, normal control, Eug (0.5 or 1 mg)- or Tam-treated rats versus breast precancerous lesion model rats (Scheff´e test).

### Effects of Eug on expression of HER2/PI3K-AKT signaling pathway key protein in breast precancerous lesion model rats

**Figure 7:**
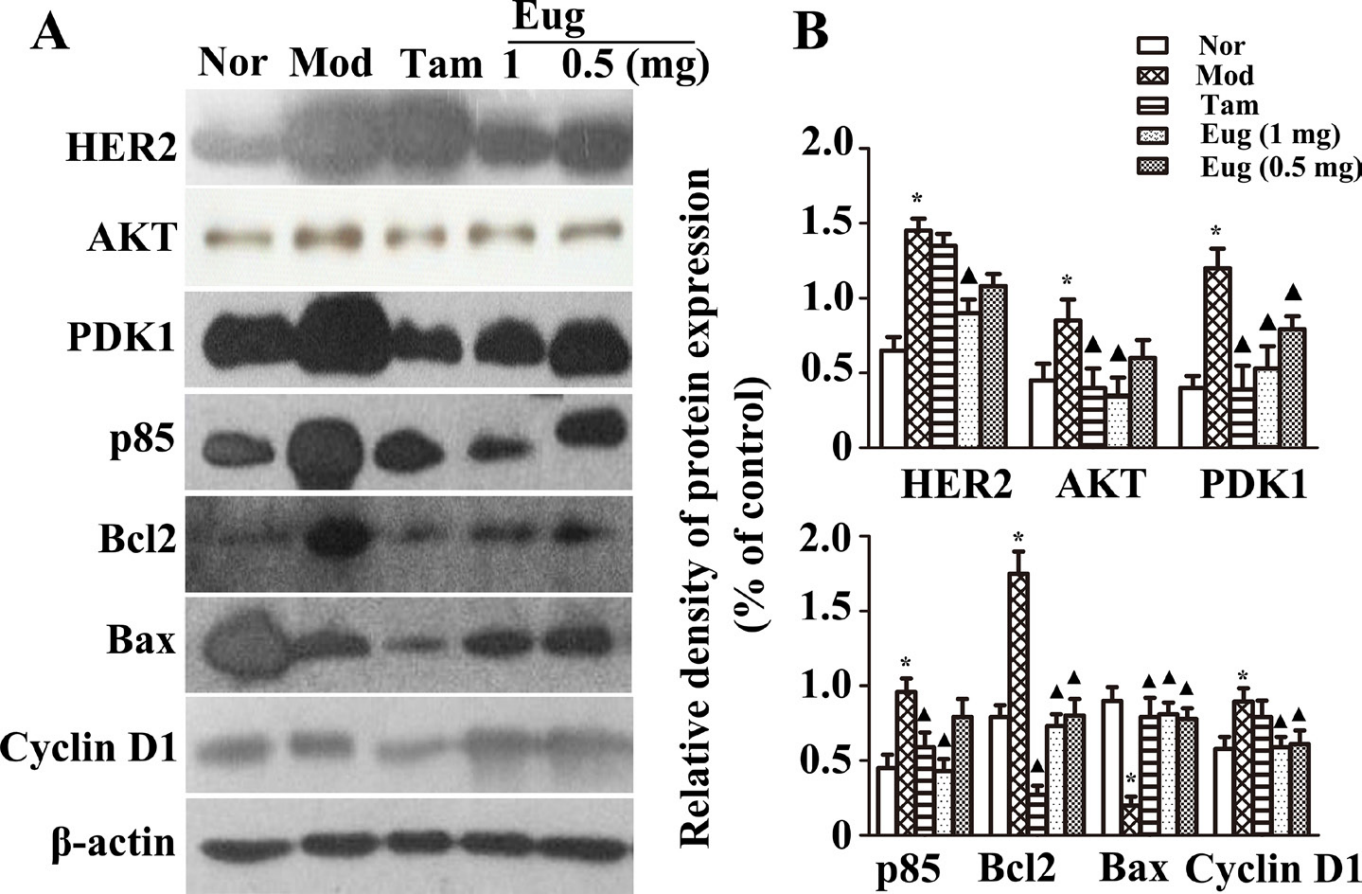
Effect of eugenol (Eug) on the key proteins expression of HER2/PI3K-AKT signaling pathway in normal (Nor) or breast precancerous lesion model (Mod) rat breast tissues (**A**) Western-blot assays for the expression of the key proteins HER2, AKT, PDK1, P85, Bcl2, Bax and Cyclin D1 in normal rats or breast precancerous lesion model rats without treatment or continuously treated with tamoxifen (Tam, 1 mg), low (0.5 mg) or high (1 mg) dose Eug for 14 weeks. (**B**) Relative expression assays for HER2, AKT, PDK1, P85, Bcl2, Bax and Cyclin D1 in (A). *P < 0.05, breast precancerous lesion model rats versus normal control rats; ▲*P* < 0.05, normal control, Eug (0.5 or 1 mg)- or Tam-treated rats versus breast precancerous lesion model rats (Scheff´e test, *n* = 12).

Breast precancerous lesion model rats were established by dimethylbenzanthracene (DMBA) combined estrogen and progesterone. To detect the therapeutic intervention effect of Eug for external use on the progression of breast precancerous lesion, from the beginning of modelling to 14th week, the rat breasts were smeared with different concentrations of Eug cream once a day. The effects of Eug on expression of HER2/PI3K-AKT signaling pathway key proteins were detected using western-blot analysis. The experiment results showed that compared with the normal blank control group, the expressions of HER2, AKT, PDK1, p85, Bcl2, and Cyclin D1 were increased by 1.27–, 0.813–, 2–, 1.136–, 1.107–and 0.786-fold respectively, and Bax protein expression was decreased by 72.9% in disease model group (Figure 7A and 7B). Compared with the disease model group, after being treated by high dose Eug cream (1 mg Eug), the expressions of HER2, AKT, PDK1, p85, Bcl-2 and Cyclin D1 protein were significantly decreased by 62.9%, 58.6%, 56.4%, 54.3%, 59.3% and 43.0% respectively, whereas Bax protein expression was increased by 2.51-fold. The expressions of these key proteins HER2, AKT and PDK1 were also significantly decreased in Tam-treated group, which were similar with the effects in high dose Eug-treated group (Figure 7A and 7B).

**Figure 8:**
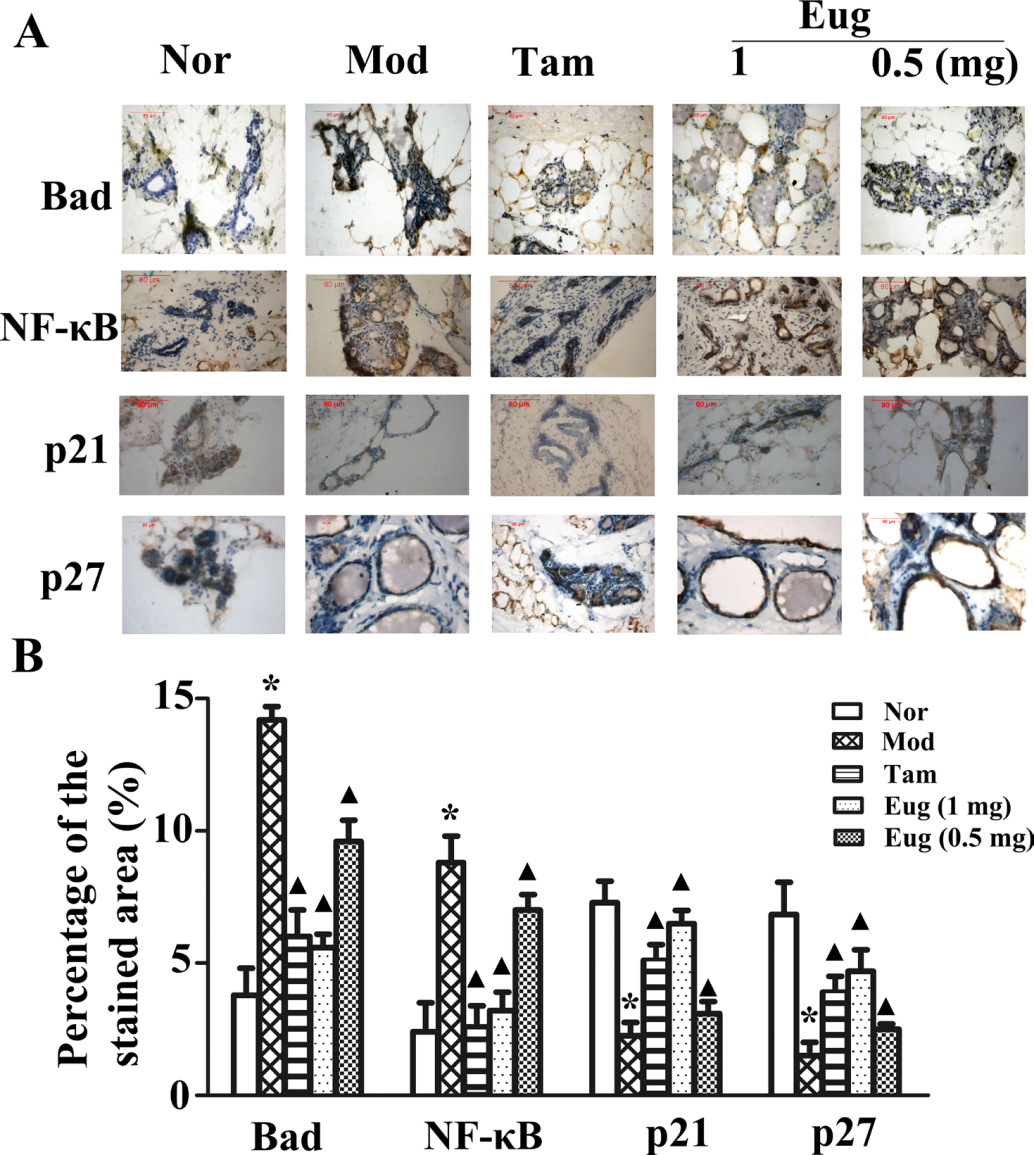
Immunohistochemical assay for the key proteins Bad, NF-κB, p21 and p27 of HER2/PI3K-AKT signaling pathway in normal (Nor) or breast precancerous lesion model (Mod) rat breast tissues without treatment or continuously treated with tamoxifen (Tam, 1 mg), low (0.5 mg) or high (1 mg) dose Eug for 14 weeks (**A**) and their relative expression assays (**B**). *P < 0.05, breast precancerous lesion model rats versus normal control rats; ▲*P* < 0.05, normal control, Eug (0.5 or 1 mg)- or Tam-treated rats versus breast precancerous lesion model rats (Scheff´e test, *n* = 12).

Immunohistochemical assay were also performed using the monoclonal antibodies including NF-κB p65 mAb, p21mAb, Bad mAb and p27 mAb. The results showed compared with the normal blank control group, the expressions of NF-κB and Bad proteins were respectively increased by 3.08- and 3.11-fold, while p21 and p27 expression was respectively decreased by 1.73- and 2.52-fold in the disease model group. Compared with the disease model group, the expressions of NF-κB and Bad proteins were significantly decreased in Tam- and Eug cream-treated groups, whereas p21 and p27 expressions were significantly increased, and Eug had a dose dependent effect. In high dose of Eug cream-treated group, the expressions of NF-κB and Bad proteins were decreased by 65.7% and 64.0%, while p21 and p27 protein expressions were increased by 1.83- and 2.52-fold, respectively (Figure 8A and 8B).

## DISCUSSION

At present, Eug has been used as antiseptic, analgesic and antibacterial agent [13]. The antiproliferative activity of Eug against melanoma, leukemia, gastric, skin tumor and prostate cancer cells has been confirmed by many research [14], and Eug can induce apoptosis in various cancer cells such as mast cells, melanoma cells and HL-60 leukemia cells [15]. HER2 is the main signal amplifier of this growth factor receptor family, HER2/PI3K-AKT signaling pathway is an important regulation pathway in the development of breast cancer [16, 17]. The current study showed that Eug had obviously therapeutic effects on breast precancerous lesions by blocking HER2/PI3K-AKT signaling transduction.

MCF10A cell is the non-tumorigenic “normal” line and exists low level of HER2 expression [18]. The MCF10-AT cell derived from xenograft-passaged MCF10-AneoT cells, generates carcinomas in about 25% of xenografts, representing the transition from normal epithelium to malignant carcinoma. HER2/ERBB2 levels were elevated in the MCF10-AT cell line. MCF-7 cell is estrogen and progesterone receptor positive luminal A breast cancer cell line, but HER2 expression is weak in MCF-7 cell [19]. BT474, a estrogen positive cell line, was isolated from a solid invasive ductal carcinoma of the breast and overexpresses HER2 [20]. Eug can significantly inhibit proliferation of the HER-2 positive breast precancerous lesion MCF-10AT cells but there was almost no significant inhibitory effect on MCF-7 or MCF-10A cells with HER2 weak expression. Furthermore the antiproliferative or apoptosis-promoting effects of Eug nearly disappeared in MCF-10AT cells with HER2 overexpression. These results indicated that HER-2 in HER2/PI3K-AKT signaling pathway might firstly mediated the antiproliferative or apoptosis-promoting effects of Eug on MCF-10AT cells. HER2 are closely related with the growth, survival, adhesion, metastasis and differentiation of breast cancer cells, and the over-expression of HER2 would increase the tyrosine kinase activity in cells, and the carcinogenic effect of HER2 mainly inhibited apoptosis and promoted tumor angiogenesis and metastasis [21]. In this study, Eug could significantly decrease HER2, p85, PDK1 and AKT protein expressions in a dose-dependent manner in MCF-10AT cells or breast precancerous lesion model rats, which could further promote cell apoptosis and inhibit tumor progression. The over-expression of PI3K regulatory subunit p85 and its interactions with tyrosine-phosphorylated receptor/adaptor proteins may make PI3K-AKT pathway keeping sustained activation, further lead to cell abnormal proliferation or apoptosis obstruction [22]. PI3K are heterodimers comprised of a regulatory subunit p85 and a catalytic subunit p110. Activation of PI3Ks may be initiated when a growth factor or ligand binds to HER2 tyrosine kinase. Thus PI3K heterodimer interacts with their intracellular portion via p85, and the activated kinase catalyses the phosphorylation of phosphatidylinositol-4,5-bisphosphate (PIP2) to phosphatidylinositol-3,4,5-triphosphate (PIP3). PIP3 acts as a docking site for AKT, a serine/threonine kinase that is the central mediator of the PI3K pathway and phosphoinositide-dependent kinase 1(PDK1). Association with PIP3 at the membrane PDK1 facilitates phosphorylation of AKT. However, the inhibition of p85 expression or activity not only induced cell apoptosis but also may counteract the over-expression of ERBB2 [20–23]. As an upstream protein of AKT, PDK1 may activate AKT and its downstream kinases. PDK1 is over-expressed in most of the cancer cells, and inhibiting the expression of PDK1 followed by decreasing the activity of AKT may become a new treatment strategy for hemangioma [21–24]. Through the phosphorylation of a diverse set of substrates including apoptosis or cell cycle regulators, activated AKT regulates cell-cycle, cell apoptosis or survival. So Eug may effectively inhibited the progress of breast precancerous lesion through blocking HER2/PI3K-AKT signaling transduction by decreasing HER2, p85, PDK1 and AKT protein expressions, inhibiting the conversion of PIP2 to PIP3 and phosphorylation of AKT.

The flow cytometry assay showed Eug could significantly promote cells apoptosis and *induce S-phase* cell cycle *arrest* in breast precancerous lesion MCF-10AT cells. Eug or Tam mainly induced the late apoptosis in MCF-10AT cells, it may be that Eug or Tam cause the DNA degradation or breakage of MCF-10AT cells so as to provoke a strong cytotoxic response [25, 26]. This study showed Eug effectively regulated the expressions of the apoptosis-related factors including NF-κB, Bad, Bcl-2, Bax and cell cycle regulators p21, p27 and cyclin D1 in breast precancerous lesion model rats. A previous study has suggested that Eug may significantly decrease the NF-κB expression in a rat model of gastric cancer, and the inhibition of NF-κB expression or activity was the important mechanism of reducing cell apoptosis [9, 27]. Some studies have showed that the expression of the Bcl-2 family proteins such as Bax, Bad and Bcl2 are closely related to the appearance and development of breast cancer [28–30]. Abnormal high expression of the Bad protein promoted prostate cancer growth, and anti-*apoptotic* molecular Bcl2 and pro-*apoptotic* molecular Bax *constitute* an apoptosis *switch* in cancer development *and* therapy [31]. The over-expression of Bcl2 not only prevents the development of cells apoptosis, but inhibits the release of cytochrome C, nevertheless, Bax may increase the membrane’s permeability, which leads to the release of cytochrome C from mitochondria, activation of caspase-9 and initiation of the caspase activation pathway for apoptosis [32, 33]. Western-blot assay showed Eug markedly decreased the protein expressions of NF-κB, Bcl2 and Bad, meanwhile significantly increased the Bax expression in breast precancerous lesion MCF-10AT cells or model rats. Thus it may be one of the important cell pro-*apoptotic* mechanisms of Eug through effectively regulating these apoptosis-related factors expressions.

Eug can also inhibit the growth of tumor cells by influencing the cells cycle [9]. This study indicated that Eug could obviously induce *S-phase* cell cycle *arrest* in breast precancerous lesion MCF-10AT cells, and reduced the protein expression of cyclinD1, meanwhile, increase P21 and p27 protein expression in breast precancerous lesion MCF-10AT cells or model rats. p21, p27, cyclin D1 and cyclin E was often associated with S-phase cell cycle arrest, and p21 and p27 are tumor suppressor proteins that retard the cell cycle progression by binding with active cyclin-CDK complexes and thereby inhibiting their activities [34–36]. Furthermore, the over-expression of CyclinD1 is parallel with the tumor rapidly growth, and abnormal high expression of CyclinD1 may cause the disorder of cell cycle and abnormal cell proliferation [37]. Especially, the expression of CDK1, CDK2, Cdc25C and Cyclin A which are mainly involved in driving the transition from S to G2/M phase were markedly reduced in HER2 non-overexpressing MCF-10AT cells after Eug treatment, which indicated Eug could inhibit the breast precancerous cells proliferation by inducing S-phase cell cycle arrest.

In brief, this study showed that eugenol external use may effectively alleviate or inhibit breast precancerous lesions through HER2/PI3K-AKT pathway-induced cell apoptosis and S-phase cell cycle arrest, indicating eugenol may be a promising external application drug to prevent or treat breast precancerous lesions.

## MATERIALS AND METHODS

### Cell lines

BT-474, a breast cancer cell line with HER-2 overexpression and breast precancerous lesion cell line MCF-10AT, and breast cancer cell line MCF-7 or MCF-10A with weak expression of HER-2 were purchased from American Karmanos cancer research institute (KCI).

The MCF-10AT cell line was monolayer adherent cell, and was cultured in a 37°C constant incubator containing 5% CO_2_. Complete media (DMEM/F-12 supplemented with 5% horse serum) was used for MCF-10AT cell culture, and the cells at logarithmic growth phase were used in the cytological experiments.

### Plasmid preparation and transfection

The coding sequence of HER2 cDNA was successfully cloned and it was consistent with the NCBI database. Then the eukaryotic expression vector pcDNA3.1-HER2 was constructed and confirmed by sequencing. The cell lines including BT-474, MCF-10AT, MCF-7 and MCF-10A with HER2 over-expression were performed by pcDNA3.1-HER2 transfection using Lipofectamine 2000 (Invitrogen, Carlsbad, CA, USA) according to the protocol of manufacturer.

### Drug preparation for rat models of breast precancerous lesions

#### Preparations of blank matrix and eugenol (Eug) cream

7 g KOH was dissolved in appropriate amount of distilled water and heated to 80°C, which was taken as the water phase. 140 g stearic acid was heated in a water bath, melted and cooled down to 80°C.100 g KOH water solution and glycerinum were added and stirred constantly, then the appropriate amount of distilled water was added and the volume was metered to 1000 ml, so as to obtain the blank matrix (Mat). The sample was continuously stirred and cooled down to 40°C. The different doses of Eug were added, thoroughly mixed and cooled down to room temperature, so as to obtain the Eug cream. Per one gram of high or low dose Eug creams respectively contained 5 or 2.5 mg Eug.

### Preparation of tamoxifen (Tam) cream

0.5 g Tam and 99.5 g blank matrix were weighed. The blank matrix was heated to melting, then Tam was slowly added under constant stirring and cooled down to room temperature. Per one gram of Tam cream contained 5 mg Tam.

### Preparation of DMBA solution

The DMBA was precisely weighed, dissolved in sesame oil according to the proportion of 7 mg/ml, placed in ultrasonic constant temperature water bath box (60°C, 40 Hz), and completely dissolved by ultrasonic oscillation.

### Effect of Eug on proliferation of the breast precancerous lesion model MCF-10AT cells and BT-474, MCF-7, MCF-10A cells

100 μl BT-474, MCF-10AT MCF-7 and MCF-10A cells suspension at logarithmic phase (5 × 10^4^ cells/ml) were respectively inoculated in each well of *96-well* culture *plate*. After being cultured for 24 h, the different concentrations of Eug medium solution [40, 80, 120, 160, 200 and 240 μM; Eug was firstly dissolved in dimethyl sulphoxide (DMSO) and then further dissolved in PBS (PH 7.4). The final concentration of DMSO in Eug medium solution was less than 0.1%, and it had no effect on cells] were added. The 50% inhibiting concentration (IC50) of Eug on MCF-10AT cells and the inhibition rates of 80 μM Eug for BT-474, MCF-10AT, MCF-7 and MCF-10A cells were detected. The PBS-treated groups were acted as the control groups (Vehicle). The sample was cultured for another 24 h. 20 μl 5 mg/ml MTT was added in every well and cultured for 4 h away from light. The cell growth inhibition rate [growth inhibition rate (%) = (1-experimental group A570/control group A570) ×100%] was detected.

In cell lines including BT-474, MCF-10AT, MCF-7 and MCF-10A with HER2 over-expression, cells were treated by the same concentration of Eug to detect the biological effect of Eug.

### Effect of Eug on MCF-10AT cells apoptosis

The MCF-10AT cells at logarithmic growth phase inoculated in 6-well plate were treated with PBS, Tam (180 μM) or Eug (180 μM) for 24 h, then the MCF-10AT cells were digested using trypsin-EDTA to obtain MCF-10AT cell suspension (5 × 10^5^ cells/ml). 1 ml MCF-10AT cell suspension was centrifuged by 1000 rpm/min at 4°C for 10 min. After the MCF-10AT cells were resuspended with 200 μl binding buffer, 10 μl Annexin V-FITC and 10 μl propidium iodide (PI) were added, and gently blended for reaction away from light for 15 min. The cell apoptosis rates were detected by the flow cytometry.

In MCF-10AT cells with HER2 overexpression, cells were treated by the same concentration of Eug (180 μM) to detect the biological effect of Eug.

### Effect of Eug on HER2 non-overexpressing and overexpressing MCF-10AT cell cycle

The MCF-10AT cells at logarithmic growth phase were inoculated with 1 × 10^5^ cells/well in a 6-well plate and cultured for 24 h. Then the cells in different groups were respectively treated with PBS, Tam (180 μM) or Eug (180 μM) for another 24 h. The cells were collected and fixed for 12 h using the pre-cooling 70% alcohol at 4°C. Then the fluorochrome PI was added and incubated at 37°C away from light for 30 min. Finally the red fluorescence was detected at 488 nm excitation wavelength by the flow cytometry, and the cell cycle distributions of MCF-10AT cells in different experimental groups were determined. In MCF-10AT cells with HER2 overexpression, cells were treated by the same concentration of Eug (180 μM) to detect the biological effect of Eug. The expression of CDK1, CDK2, Cdc25C and Cyclin A were detected by western-blot analysis as described previously [38].

### Effect of Eug on HER2/PI3K-AKT signaling pathway key proteins expressions in HER2 non-overexpressing and overexpressing MCF-10AT cells

After MCF-10AT cells in logarithmic growth phase were treated with Tam (180 μM), Eug, PBS or blank matrix (Mat) for 24 h, HER2 protein expressions were assayed (the concentrations of Eug are 100, 140 and 180 μM), and the HER2/PI3K-AKT signaling pathway key proteins expressions including PDK1, p85, AKT, NF-κB, Bad, Bcl-2, Bax, cyclin D1, p21 and p27 (the concentration of Eug is 180 μM) were detected by western-blot analysis as described previously [38]. In MCF-10AT cells with HER2 over-expression, cells were treated by the same concentration of Eug (180 μM) to detect the biological effect of Eug.

### Establishment of breast precancerous lesion rat model

Sixty healthy female six-week-old SD rats with weight 160 g-180 g (SPF, license number: SCXK 2013-0002) were fed in animal laboratory management center SPF grade animal room (indoor temperature 25°C, natural lighting, free diet and regular disinfection) in Jinan University. After environmental adaptation for one week, the rats hair around the breast was removed (1 cm diameter with the nipple as the center). The breast precancerous lesion SD rat model was induced using DMBA in combination with estrogen and progesterone as described previously [39–41]. The rats were randomly divided into 5 groups according to their weight (12 per group): normal blank control group, disease model group, Tam-treated group, high dose Eug-treated group and low dose Eug-treated group. The rats were sacrificed and assayed in the 14th week.

### Therapeutic intervention effect of Eug for external use on breast precancerous lesion model rats

#### Normal blank control group (Nor)

The rats received one-time gavage using 1 ml sesame oil/(100 g body weight) and were conventionally fed for 14 weeks.

### Breast precancerous lesion model group (Mod)

The rats received one-time gavage using 1ml DMBA sesame oil/(100 g body weight). The next five days acted as a cycle (5-day cycle), and 0.5 mg benzoate estradiol/(kg body weight) was injected in the medial muscles of hind legs from the first day to the third day. Then 4 mg progesterone/(kg body weight) was injected on the fourth day. The rats were observed on the fifth day. Continuous 12 cycles (5-day cycle) were performed. The rats were conventionally fed for 14 weeks.

### Tam group

Based on the method of the disease model group, from the first day of the first 5-day cycle to the end of the 14th week, 0.2 g Tam cream (containing 1 mg Tam) was smeared and massaged for 1 min on the breast of every rat once a day. The rats were conventionally fed for 14 weeks.

### High dose Eug group

Based on the method of the disease model group, from the first day of the first 5-day cycle to the end of the 14th week, 0.2 g high dose Eug preparation (containing 1 mg Eug) was smeared and massaged for 1 min on the breast of every rat once a day. The rats were conventionally fed for 14 weeks.

### Low dose Eug group

Based on the method of the disease model group, from the first day of the first 5-day cycle to the end of the 14th week, 0.2 g low dose Eug preparation (containing 0.5 mg eugenol) was smeared and massaged for 1 min on the breast of every rat once a day. The rats were conventionally fed for 14 weeks.

### Western-blot detection

*The* animal *experiments* were terminated *at* *the* *end* *of* the *14th week, and* RIPA Lysis Buffer (Beyotime Biotechnology, Shanghai, China) was used to extract the total protein of rat breast tissue in different groups. The total protein was separated by 12% sodium dodecyl sulfate polyacrylamide gel electrophoresis (SDS-PAGE) and transferred onto polyvinylidene difluoride membranes (Millipore, Billerica, USA). The membranes were respectively incubated with the HER2 Rabbit mAb, AKT Rabbit mAb, PI3 Kinase p85 Rabbit mAb, Cyclin D1 Rabbit mAb, Bax antibody, Bcl-2 Rabbit mAb, PDK1 Rabbit mAb(Cell Signaling Technology, Inc., Boston, USA), NF-κB p65 mAb, p21 mAb-Bad mAb and p27 mAb (Santa Cruz Biotechnology, CA, USA) overnight at 4°C. The horseradish peroxidase (HRP)-conjugated goat-anti-rabbit IgG (Cell Signaling Technology, Inc., Boston, USA) was used as the second antibody. Protein bands were visualized by using an ECL kit (Santa Cruz Biotechnology) and densitometric analysis of Western blots was performed with Image J analysis software.

### Immunohistochemical detection

Establishment of breast precancerous lesion rat model and the animal experiments were performed after the approval from the Laboratory Animal Ethics Committee of Jinan University. Animal welfare and experimental procedures were carried out in accordance with the Guide for the Care and Use of Laboratory Animals (Ministry of Science and Technology of China, 2006) and related ethical regulations of Jinan University.

After the animal experiments were terminated at the end of the 14th week, the paraffin sample of rat breast tissues in different experimental groups were serially sectioned (4 μm), conventionally baked, dewaxed and hydrated. The routine immunohistochemical streptavidin-peroxidase (SP) procedures were conducted [42]. The protein expression was observed under light microscope. The positive cells expression average area percentage was analyzed using Leica Qwin software. The statistical analysis was conducted.

### Statistical analysis

Results are presented as mean ± SEM of at least three independent experiments. Differences between groups were analyzed using analysis of variance with SPSS version 15.0 (International Business Machines Corporation, Armonk, New York, USA). Post-hoc analysis was used if the analysis of variance was significant. *P* < 0.05 indicated that the difference was statistically significant.

## Abbreviations

HER2: Human epidermal growth factor receptor 2
ER: Estrogen receptor
ERBB2: v-erb-b2 avian erythroblastic leukemia viral oncogene homolog 2
PR: Progesterone receptor
PDK1: 3-Phosphoinositide-dependentprotein kinase-1
NF-κB: nuclear factor-κB
Bad: Bcl-2 antagonist of cell death
Bcl-2: B cell lymphoma-2
Bax: Bcl-2-associated X protein
BSLT: brine shrimp lethality test
MTT: 3-(4,5-Dimethylthiazol-2-yl)-2,5-Diphenyltetrazolium Bromide
KCI: Karmanos cancer research institute
Eug: eugenol
Mat: matrix
Tam: tamoxifen
DMBA: dimethylbenzanthracene
DMSO: dimethyl sulphoxide
PI: propidium iodide
Nor: Normal blank control group
Mod: Disease model group
SP: streptavidin-peroxidase.
